# Comparison of Vegetable Waste Byproducts of Selected Cultivars of *Foeniculum vulgare* Mill. by an Integrated LC‐(HR)MS and ^1^H‐NMR‐Based Metabolomics Approach

**DOI:** 10.1002/pca.3488

**Published:** 2025-01-21

**Authors:** Maria Assunta Crescenzi, Antonietta Cerulli, Milena Masullo, Paola Montoro, Sonia Piacente

**Affiliations:** ^1^ Department of Pharmacy University of the Study of Salerno Fisciano Italy; ^2^ Ph.D. Program in Drug Discovery and Development, Department of Pharmacy University of the Study of Salerno Fisciano Italy; ^3^ National Biodiversity Future Center (NBFC) Palermo Italy

**Keywords:** ^1^H‐NMR and HRMS‐based metabolomics, *Foeniculum vulgare*, multivariate data analysis, vegetable waste

## Abstract

**Introduction:**

The metabolome of plants is influenced by various factors, especially environmental, as the season in which they are grown. So, distinct varieties of the identical plant might show an increase or decrease in metabolites. The diversity of content of primary and secondary metabolites can also determine the variation in their biological properties. Due to the current occurrence of various fennel varieties, the crop can now be grown for the entire year.

**Objective:**

This work used an integrated approach of LC/MS and NMR analysis to characterize the metabolome of fennel waste of different varieties by multivariate statistical analysis.

**Methods:**

The extracts were investigated by NMR and LC/MS analysis to focus attention on the primary and secondary metabolites. Both LC‐HRMS and NMR data were analyzed by principal component analysis (PCA).

**Results:**

The ^1^H‐NMR analysis led to the identification of 15 primary metabolites, such as amino acids, carbohydrates, and organic acid derivatives. The secondary metabolites identified by LC/MS analysis mainly belong to the phenolic, lipid, and fatty acid compounds classes.

**Conclusion:**

This integrated approach guarantees a precise and complete overview of the variations in the metabolic expression of the fennel varieties grown in different seasons.

## 1 Introduction

The plant metabolome includes primary and secondary metabolites. The synergistic relationship between primary and secondary metabolites enables plants to optimize their growth, defense, and adaptation to changing environmental conditions. Primary metabolites provide the foundational resources—energy, building blocks, and regulatory signals—necessary for life processes. Secondary metabolites, while not essential for basic growth, enhance the plant's ability to survive and thrive in a competitive, stressful environment. By working together, these metabolites form a highly integrated metabolic network that supports both the plant's fundamental life functions and its ability to produce specialized compounds for defense, reproduction, and communication with other organisms. This synergy is crucial not only for plant health but also for the nutritional and therapeutic potential of plant‐based products used in food, medicine, and industry [1]. Metabolites produced by plants are not stable over time. Several elements influence the metabolic expression of the plant kingdom, such as genetic, morphogenetic, and environmental factors [2].

Specifically, environmental factors may affect the production and collection of metabolites in plants. Among these factors, some relate to soil attributes, such as traces of metals and pH, as well as seasonality changes, such as drought, cold, heat, and ultraviolet radiation. The response to these alterations is different from species to species, and accordingly, the content of both primary and secondary metabolites can vary [3]. Metabolomics allows us to evaluate the metabolite profile of plants in response to environmental alterations such as seasonality [4].

This work aimed at characterizing waste metabolomes of three varieties of *Foeniculum vulgare* Mill. The choice of this marketed plant matrix was based on its “IV gamma” agricultural food product classification, which is known to produce large quantities of plant waste in southern Italy. Moreover, fennel is cultivated the entire year in Italy because respective varieties are adapted to specific seasonal conditions. *F. vulgare* , which belongs to the Apiaceae family, has little stems that divide into superficial leaves of an intense green‐blue color [5]. The present study focuses on two plant parts of fennel waste byproducts that include the leaves and little stems from three seasonal varieties: Tiziano (winter), Pegaso (spring), and Preludio (summer).

Our research group has developed methods to characterize and identify biomarker secondary metabolites found in fennel waste products [6]. We focused on four waste parts: the bulb, the superficial leaves, the main stem and the little stems. Using various extraction techniques, metabolites belonging mostly to the classes of phenolic acids, glycosylated flavonoids and iridoids were identified. After characterizing the various waste byproducts, an attempt was made to understand which part of the waste material was the richest in the content of bioactive compounds with promising biological activities. So, these metabolites were quantified using a targeted approach, revealing that the leaves and little stems are richest in bioactive compounds [7]. In our ongoing investigation, the study is extended to different fennel varieties and focuses on metabolites identified in previous work on these parts evidenced as richest in metabolites [8].

This study aims to expand and complete the primary and secondary metabolic characterizations in fennel waste products of different cultivars of *F. vulgare* by combining spectral datasets obtained from mass spectrometry coupled with liquid chromatography (LC–MS) and nuclear magnetic resonance spectroscopy (NMR) [9, 10]. The integrated approach between the two techniques provides complementary and additional results while it guarantees a more comprehensive and complete assessment of the metabolome of a plant species [11].

An analytical approach has been previously published to characterize secondary metabolites from fennel waste products of Tiziano variety [6]. This approach combined LC‐ESI/LTQ‐Orbitrap‐MS and MS/MS datasets for Multivariate Data Analysis. At the same time, NMR spectroscopy, a nondestructive spectroscopic technique that permits a fast analysis by simple sample preparation, could afford complete fingerprints of plant extracts.

In recent years, a challenge in metabolomics approaches for plant studies is the possibility of developing fused dataset protocols based on MS and NMR [12, 13]. In few studies, the application of fused datasets to metabolomics analyses resulted in more robust statistical models. Furthermore, the fusion technique on MS/NMR‐based datasets can give additional interpretation of the generated analytical results.

The innovative approach is to fuse NMR and LC–MS spectral datasets for a comprehensive assessment of the metabolome of fennel waste products from cultivars grown in different geographical areas and seasons.

## 2 Materials and Methods

### 2.1 Raw Materials

The fennel ( *F. vulgare* ) waste byproducts were recovered at three separate times of the year, as described in our previous work [8]. The Tiziano variety was harvested in December 2019 and the Pegaso variety in April 2021. Both Tiziano and Pegaso cultivars were grown in Molise region (Campomarino). Preludio variety, grown in Abruzzo region (Avezzano), was harvested in July 2021. The vegetable waste byproducts consisted of superficial leaves and little stems. The sample codes used were FVLS_T ( *F. vulgare* little stems of variety Tiziano), FVLE_T ( *F. vulgare* leaves of variety Tiziano), FVLS_PE ( *F. vulgare* little stems of variety Pegaso), FVLE_PE ( *F. vulgare* leaves of variety Pegaso), FVLS_PR ( *F. vulgare* little stems of variety Preludio), and FVLE_PR ( *F. vulgare* leaves of variety Preludio).

### 2.2 Sample Preparation

The fennel waste byproducts were frozen at −80°C and then extracted with 80% hydroalcoholic solution assisted by ultrasound, as previously described [8]. More details on the extraction protocol can be found in the Supporting Information. In an ultrasonic bath, the extraction was done using 1 g of little stem or leaf with 40 mL of ethanol/water (80:20) for 15 min. The extraction was repeated three times, and the extracts were filtered with filter paper 67 g/m^2^ (530‐16100‐Aptaca). For LC–MS analysis, the extracts, dried under a nitrogen stream, were dissolved in methanol with a final concentration of 1 mg/mL.

### 2.3 LC‐ESI/LTQ‐Orbitrap/MS Analysis

LC‐ESI/LTQOrbitrap/MS analyses were performed to identify secondary metabolites in waste byproducts of three fennel varieties (Supporting Information). A Kinetex EVO 5.0‐μm column (150 mm × 2.1 mm) was used. Mobile phases utilized for chromatographic separation were water + 0.1% formic acid (A) and acetonitrile + 0.1% formic acid (B). To study the fragmentation and to characterize the fennel metabolites, a data‐dependent scan was set up. The analyses were conducted in the negative ion mode, and the data were processed with Xcalibur 2.1 (Supporting Information). Metabolite characterization was done by studying fragmentation patterns, using databases such as MassBank, Knapsack and FoodB, and a literature study.

### 2.4 Pseudo‐Targeted Multivariate Statistical Analysis of the Secondary Metabolites by LC‐(HR)MS Approach

Multivariate statistical analysis was used to define differences in the metabolome of the samples under investigation. A previously published pseudo‐targeted approach was used [14]. For the multivariate statistical analysis, five biological replicates were prepared, and each replicate was analyzed twice as technical duplicates. The LC‐ESI/LTQOrbitrap/MS chromatograms, analyzed in full scan, were processed with the software MZmine 2.38 (http://mzmine.sourceforge.net), which generated an aligned and normalized peak list. The noise level threshold was set to 1.0 × 10^4^, excluding all lower‐intensity peaks from the analysis. A data matrix was created whose rows represent the observations, that is, each of the different replicates of the samples (*n* = 60), and whose columns represent the variables, that is, the areas of the peaks associated with the *m/z*. Using a pseudo‐targeted approach, all nonidentified *m/z* were removed from the data matrix. The final data matrix contained 25 variables. The dataset created was processed with SIMCA 12.0 (Umetrix AB, Umea Sweden), to conduct a PCA, an unsupervised technique to simplify the data and to define chemical markers for each sample. The dataset was scaled to Unit Variance.

### 2.5 NMR Analysis and Data Processing

NMR analyses were performed on a Bruker‐500 spectrometer. For ^1^H‐NMR analysis, the extracts were dissolved in 550 μL of methanol‐*d*_*4*_ and after 5 μL of TSP was added to attain a final concentration of 4.88 mM and transferred into a 5 mm of NMR tube. TSP was used as an external reference (reference peak set at 0 ppm). Each sample was prepared in duplicate. Three experiments were carried out on each sample. All samples were run at 300 K, using the zgesgp pulse sequence specific parameters (Supporting Information); fennel extracts were further analyzed by 2D‐NMR experiments including DQF‐COSY, HSQC, and HMBC, and TOCSY. After the acquisition, ^1^H‐NMR spectra were manually processed by MestreNova 10 software (Supporting Information), Bucketing was performed within the −0.5‐8.5 ppm region (bin width of 0.04 ppm), excluding the signals of the residual nondeuterated methanol and deuterated methanol. Pareto scaling was applied before PCA; the matrix was built by 15 variables (specific chemical shift of primary metabolites), and 12 observations (extracts).

### 2.6 Pseudo‐Targeted Multivariate Statistical Analysis of MS‐NMR Fused Data

A new data matrix was created to assess the expression of primary and secondary metabolites within the fennel different cultivars. The dataset comprised of 36 observations (samples) and 52 variables (primary and secondary metabolites), and it was processed with SIMCA 12.0 (Umetrix AB, Umea Sweden). To assess metabolites interaction with the clusters of samples, the supervised analysis of partial least squares discriminant analysis‐regression (PLS‐DA) was conducted. The new dataset was scaled with Pareto algorithm. Block 1/sqrt scaling was utilized for the merged datasets, allowing each block of variables (NMR and MS) to be evaluated as a unit and given the corresponding variation. Numerical values were assigned to the sample categories: −1, Tiziano variety; 0, Pegaso variety; +1, Preludio variety.

## 3 Results and Discussion

### 3.1 Identification of Metabolites of two Plant Parts of Three Varieties and Parts of *F. vulgare* Waste Byproducts by LC‐ESI/LTQ‐Orbitrap Full Scan and MS/MS Analysis

Metabolite profiling of hydroalcoholic extracts of fennel waste byproducts enabled the identification of 44 secondary metabolites (Table 1). The spectral dataset acquired by the LC‐ESI/LTQ‐Orbitrap/MS/MS, which was used to determine the accurate mass at ≤ 5 ppm, was processed using the Xcalibur software. By examining fragmentation and using databases such as Knapsack, FoodB, and MassBank, putative identifications could be assigned. Crescenzi et al. [6] previously characterized metabolites on different plant parts of fennel waste byproducts, focusing exclusively on the Tiziano variety.

**TABLE 1:** Metabolites identified in little stem and leaf extracts of *Foeniculum vulgare* waste byproducts by LC‐ESI/LTQ‐Orbitrap full scan and MS/MS analysis.

No.	RT	[M‐H]^−^	Molecular formula	Δppm	MS/MS	Identity	FVLS_T	FVLE_T	FVLS_PE	FVLE_PE	FVLS_PR	FVLE_PR	Reference
**1**	6.42	343.10312	C_15_H_19_O_9_	2.2	181.0/328.1	4‐glucopyranosyloxy‐3‐methoybenzeneacetic acid	✔	✔			✔		[6]
**2**	8.85	325.09268	C_15_H_17_O_8_	2.7	163.0	*p*‐coumaric acid glucoside				✔	✔	✔	[15]
**3**	9.74	353.08759	C_16_H_17_O_9_	0.9	191.1	Chlorogenic acid	✔	✔	✔	✔	✔	✔	[6]
**4**	10.58	210.07710	C_10_H_12_O_4_N	2.4	124.0	Methyldopa	✔	✔					[15]
**5**	10.99	583.20209	C_27_H_35_O_14_	−1.0	375.1/537.2/327.1	Lucidumoside C	✔	✔			✔	✔	[6]
**6**	11.10	433.20715	C_20_H_33_O_10_	0.7	387.2	Unknown				✔		✔	[6]
**7**	12.81	371.09756	C_16_H_19_O_10_	0.8	249.1	Deacetylasperuloside		✔					[6]
**8**	13.38	367.10318	C_17_H_19_O_9_	2.2	191.1	3‐*O*‐feruloyl quinic acid	✔	✔		✔	✔	✔	[6]
**9**	14.37	523.21808	C_26_H_35_O_11_	1.3	361.2	Secoisolariciresinol 9‐glucoside					✔		[6]
**10**	14.91	425.14456	C_20_H_25_O_10_	0.8	263.1	2H‐1‐benzopyran‐6‐propanoic acid, 7‐(*β*‐D‐glucopyranosyloxy)‐3,4‐dihydro‐2,2‐dimethyl‐4‐oxo—		✔				✔	[6]
**11**	15.77	341.12384	C_16_H_21_O_8_	0.6	179.1/135.1	Sphalleroside A	✔						[16]
**12**	15.81	473.20209	C_22_H_33_O_11_	−0.4	429.2	Unknown				✔		✔	
**13**	16.46	389.12375	C_20_H_21_O_8_	−0.3	341.1/193.1	Resveratrol 3‐*O*‐glucoside	✔						[6]
**14**	16.75	609.14514	C_27_H_29_O_16_	0.2	301.0	Quercetin‐3‐*O*‐rutinoside					✔	✔	[6]
**15**	17.39	463.08768	C_21_H_19_O_12_	0.1	301.0	Quercetin 3‐*O*‐glucoside		✔		✔	✔	✔	[6]
**16**	18.47	477.06656	C_21_H_17_O_13_	−1.5	301.0	quercetin‐*O*‐glucuronide	✔	✔	✔	✔	✔	✔	[6]
**17**	18.83	447.09253	C_21_ H_19_ O_11_	−0.5	284.0/327.0	Kaempferol‐3‐*O*‐glucopyranoside				✔			[6]
**18**	19.16	187.09752	C_9_H_15_O_4_	2.4	125.1	Azelaic acid	✔	✔					[15]
**19**	20.13	515.11865	C_25_H_23_O_12_	−0.6	353.1/191.1	Dicaffeoyl quinic acid	✔	✔			✔		[6]
**20**	20.16	461.07199	C_21_H_17_O_12_	0.2	285.1	Luteolin‐7‐*O*‐glucuronide		✔		✔		✔	[6]
**21**	21.14	359.07666	C_18_H_15_O_8_	−0.1	161.0	Rosmarinic acid	✔		✔				[17]
**22**	21.49	601.11908	C_28_H_25_O_15_	−0.6	395.1/557.1/515.1/439.1	Malonyl‐1,4‐*O*‐dicaffeoylquinic acid	✔	✔			✔	✔	[6]
**23**	21.64	413.21765	C_21_H_33_O_8_	0.2	353.2	Unknown				✔			
**24**	21.67	299.18604	C_16_H_27_O_5_	0.6	201.1/183.1/263.2	1‐14 dimethyl‐2‐oxotetradecanedioate	✔						[6]
**25**	23.26	407.10086	C_12_H_23_O_15_	−5.6	241.0/392.1	Unknown		✔					
**26**	23.50	405.08539	C_12_H_21_O_15_	−5.2	257.0/241.0/390.1	Unknown		✔					
**27**	24.25	337.12900	C_17_H_21_O_7_	0.8	133.1	Unknown				✔	✔	✔	
**28**	25.07	491.28543	C_22_H_19_O_13_	−0.4	315.1	Isorhamnetin‐3‐*O*‐*β*‐D‐glucuronide	✔	✔	✔	✔			[6]
**29**	28.40	327.21735	C_18_H_31_O_5_	2.3	291.2/309.2/183.1/211.1/229.1	Trihydroxyoctadecaedienoic acid	✔	✔	✔	✔	✔	✔	[6]
**30**	31.44	287.22260	C_16_H_31_O_4_	1.3	269.2/241.2	10,16‐dihydroxyhexadecanoic acid				✔			[16]
**31**	33.37	329.23300	C_18_H_33_O_5_	0.6	171.1/201.1/275.2/311.2/293.2	Trihydroxyoctadecanoic acid	✔	✔	✔	✔	✔	✔	[6]
**32**	35.93	573.29114	C_28_H_45_O_12_	0.1	341.2/323.1/249.2	Unknown				✔	✔		
**33**	36.03	293.17572	C_17_H_25_O_4_	3.3	236.1/221.2	6‐gingerol		✔	✔	✔	✔	✔	[6]
**34**	36.33	311.22253	C_18_H_31_O_4_	2.7	293.2/223.2/275.2/235.2	12,13‐DiHODE (9,15)^a^	✔		✔				[18]
**35**	36.52	721.36450 [(M + FA)‐H]^−^	C_34_H_57_O_16_	−0.2	675.4	Gingerglycolipid A		✔	✔	✔	✔	✔	[6]
**36**	37.24	325.18417	C_18_H_29_O_3_S	3.0	183.0/197.0	Dodecylbenzenesulfonic acid	✔	✔	✔				[19]
**37**	37.50	601.32239	C_30_H_49_O_12_	0	341.1/323.1/277.2	Unknown				✔			
**38**	37.53	313.23810	C_18_H_33_O_4_	0.7	201.1/295.2/277.2/171.1	9,10‐DiHOME (12)^b^	✔		✔				[15]
**39**	38.17	559.31213 [(M + FA)‐H]^−^	C_28_H_47_O_11_	0.5	513.3	MGMG (18:3)^c^				✔			[20]
**40**	38.52	540.33008 [(M + FA)‐H]^−^	C_24_H_50_O_7_NP	0.5	480.3	l‐PC (16:0)^d^	✔						[20]
**41**	39.00	293.21176	C_18_H_29_O_3_	2.2	275.2/235.2	Hydroxyoctadecatrienoic acid	✔		✔	✔			[16]
**42**	39.78	291.19632	C_18_H_27_O_3_	2.9	247.2/273.1	Hydroxyoctadecatetranoic acid	✔		✔				[21]
**43**	39.78	309.20691	C_18_H_29_O_4_	2.8	291.2	Hydroperoxyoctadecatrienoic acid	✔		✔				[22]
**44**	40.03	295.22769	C1_8_H_31_O_3_	1.2	277.2/171.1	9‐HODE (10,12)^e^	✔		✔				[16]

^a^ 12,13‐dihydroxyoctadeca‐9,15‐dienoic acid.

^b^ 9,10‐dihydroxyoctadec‐12‐enoic acid.

^c^ 1‐o‐alpha‐linolenoyl‐3‐o‐beta‐galactopyranosyl‐sn‐glycerol.

^d^ 1‐Palmitoyl‐sn‐glycero‐3‐phosphocholine.

^e^ 9‐hydroxyoctadeca‐10,12‐dienoic acid.

The extracts obtained from the little stems and leaf waste byproducts of three different fennel varieties were mainly rich in phenolic acids (**1**, **3**, **8**, **19**, and **22**), glycosylated flavonoids (**14–17**, **20**, and **28**), polar lipids (**35**, **39**, and **40**), and fatty acids (**24**, **29–31**, **34**, **38**, and **41–44**).

**FIGURE 1:**
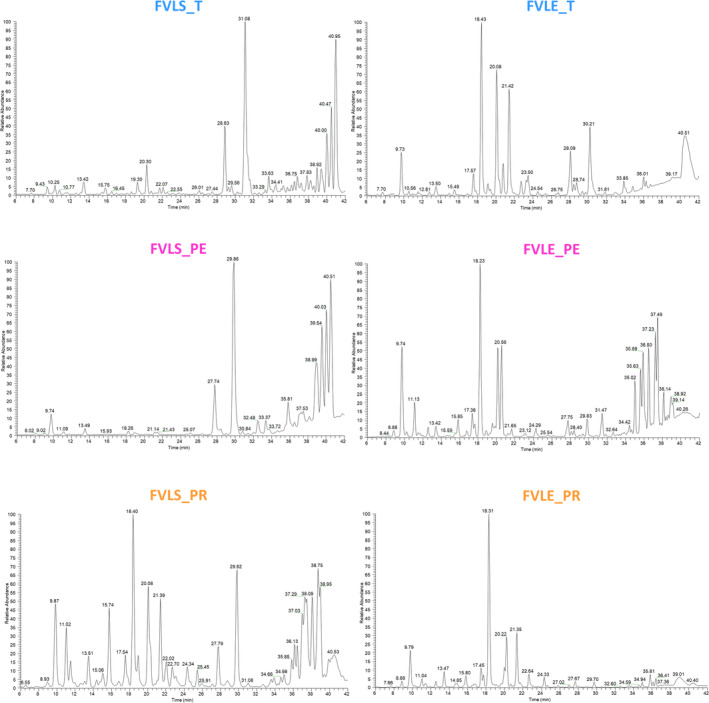
Profiles of three varieties of *Foeniculum vulgare* little stem and leaf extracts registered by LC‐ESI/LTQ/Orbitrap/MS/MS analysis (negative ion mode).

The metabolite profiles of little stem extracts were almost similar except for the FVLS‐PR sample, which presented more intense TIC peaks (Figure 1). In addition, little stem extracts of all varieties along with Pegaso leaf extract showed intense peaks between the retention time of 31 and 38 min. The metabolites identified in this retention time range were lipids and fatty acids. This is in agreement with the greater antioxidant activity, attributable to flavonoids, of fennel leaf extracts [8].

There are certain secondary metabolites in fennel waste byproducts that occurred in all three varieties as well as in both plant parts studied. These metabolites included chlorogenic acid, quercetin‐*O*‐glucuronide, and the two oxylipins trihydroxy‐octadecaedienoic acid and trihydroxy‐octadecanoic acid.

The comparison of the stem and leaf extracts from three fennel varieties harvested at different seasonality showed metabolites that were unique to the Tiziano variety. In particular, metabolites **13** and **24** with parent ion at m/z 389.12375 and 299.18604 were identified as resveratrol 3‐*O*‐glucoside and 1,14‐dimethyl‐2‐oxotetradecanedioate respectively.

It is known that seasonal variations can influence biosynthesis and flavonoid content within the plant [23]. Such changes within plants grown in different seasons naturally result in their different biological activity. There is a strong correlation between plant chemical composition and bioactivity, which varies from season to season, and metabolic and climatological factors including temperature, soil moisture, and precipitation. Drought, heat, and light influence the levels of flavonoids and phenolics in plants. Indeed, Shi et al. showed an increase in flavonoids in *Tetrastigma hemsleyanum* plants grown in the spring season.

In this work, infact, the glycosylated flavonoid kaempferol‐3‐*O*‐glucopyranoside was detected only in the little stem of the Pegaso spring variety, with the pseudo molecular ion peak at *m/z* 447.09253 [M‐H]^−^, which produced the fragment ions at *m/z* 284.0 and 327.0. By MS/MS analysis, the presence of quercetin‐3‐*O*‐rutinoside was detected only in the Preludio waste byproducts but in both of its plant parts. On the other hand, the flavonoid luteolin‐7‐*O*‐glucuronide was exclusively found in the leaf waste byproducts of the three fennel varieties. Alternatively, fatty acid metabolites (**34**, **38**, **42** and **43**) were found only in little stem extracts of *F. vulgare* , which also included 12,13‐DiHODE (**9**,**15**), 9,10‐DiHOME (**12**), hydroxyoctadecatetranoic acid (**42**), and hydroperoxyloctadecatrienoic acid (**43**). Additionally, glycerophospholipid l‐PC (16:0) (**40**) also belongs to the metabolites that only occur in the little fennel stem samples.

### 3.2 Pseudo‐Targeted Multivariate Statistical Analysis of Secondary Metabolites by LC‐(HR)MS Approach

The total ion chromatograms obtained from sample extracts of three fennel varieties were subjected to PCA. Raw data were processed with MZmine (http://mzmine.sourceforge.net/) to generate a matrix with rows representing 60 analyzed samples and columns for integrated and normalized peak areas for 37 annotated metabolites.

This analysis was aimed at identifying secondary metabolites as potential chemical markers for the various plant parts of the waste byproducts of respective varieties being investigated in this study. The PCA model generated by multivariate statistical analysis showed the first component at a variance of 45% while the second component was at 20%.

**FIGURE 2:**
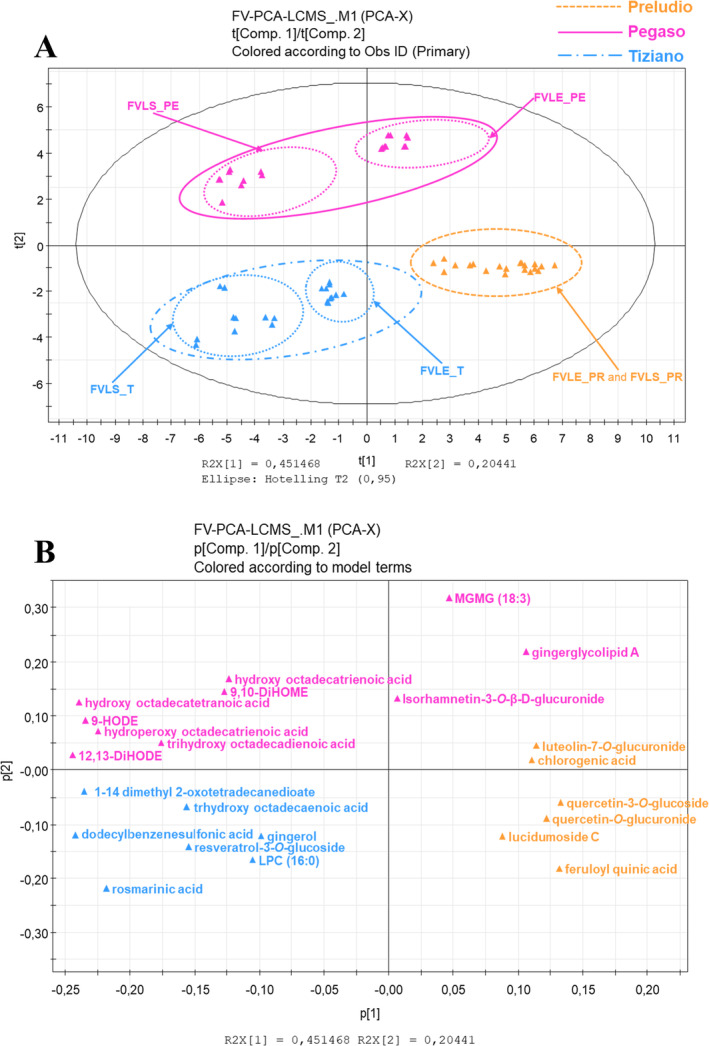
PCA scores (A) and loadings (B) scatter plot of secondary metabolites generated from the LC‐ESI‐LTQ‐Orbitrap‐MS pseudo‐targeted analysis of little stem and leaf extracts of fennel waste byproducts.

The PCA scores scatter plot (Figure 2A) certainly gave a good clustering of samples belonging to each of the three respective varieties while a good separation of samples was observed between varieties. The fennel plant samples collected from different seasons indeed afforded three well‐distinguishable groups in the MVA scatter plot. In particular, the varieties Tiziano (winter) and Preludio (summer) were situated in the lower quadrants of the scores plot, while the Pegaso (spring) variety occupies the upper quadrants. This could indicate a more similar metabolic expression of the summer and winter varieties compared to the spring varieties. In the scores scatter plot, the spring variety Pegasus stands out from the other two varieties of different seasonality. This could be related to the fact that in spring there is a higher production of secondary metabolites whose production prefers milder climatic conditions. Additionally, a cluster separation was also observed between the little stem and leaf extracts of the Pegaso and Tiziano varieties, indicating differences in metabolic content between the two plant parts.

The loadings plot (Figure 2B) highlighted the more relatively abundant annotated metabolites responsible for discriminating the various extracts of *F. vulgare* into respective clusters as observed in the scores plot.

The metabolites mostly expressed from the leaf extracts of the Pegaso variety were MGMG lipid (18:3) (**39**) and gingerglycolipid A (**35**) and the flavonoid isorhamnetin‐3‐*O*‐*β*‐D‐glucuronide (**28**). In contrast, the little stem extract of the spring variety is particularly rich in fatty acids such as hydroxyoctadecatrienoic acid (**41**), hydroxyoctadecatetranoic acid (**42**), hydroperoxyoctadecatrienoic acid (**43**), trihydroxyoctadecadienoic acid (**29**), 9,10‐DiHOME (**38**), 9‐HODE (**44**), and 12,13‐DiHODE (**34**). The low temperatures typical of the winter season induce a decrease in total membrane lipid content [24]. This could explain why the identified fatty acids appear to be chemical discriminants for the spring variety Pegaso.

For the Tiziano variety, the phytochemicals of significant abundance are gingerol (**33**), 1–14‐dimethyl 2‐oxotetradecanediote (**24**), trihydroxy octadecaenoic acid (**31**), dodecylbenzenesulfonic acid (**36**), resveratrol‐3‐*O*‐glucoside (**13**), l‐PC (16:0) (**40**), and rosmarinic acid (**21**). The presence of rosmarinic acid among the chemical markers of the Tiziano winter variety is in agreement with data already present in the literature [25]. Infact, the accumulation of rosmarinic acid depends not only on extrinsic factors but also on two key enzymes, PAL (phenylalanine ammonia‐lyase) and HPR (4‐hydroxyphenylpiruvate reductase tyrosine), for the regulation of its metabolic pathway. PAL and HPR are activated under conditions of reduced solar radiation and reduced water stress, which are characteristic of winter, which could explain the higher content of this compound in this season. Concerning resveratrol‐3‐*O*‐glucoside, for example, the data in the literature are more discordant, as in some plants there is an increase in resveratrol‐3‐*O*‐glucoside in winter and others a decrease [26]. This suggests that the increase of this compound in the Tiziano variety is not related to the season but directly to the variety of the plant.

For the summer‐grown Preludio variety, it was found to be the richest in flavonoids such as quercetin‐*O*‐glucuronide (**16)**, quercetin‐3‐*O*‐glucoside (**15**), luteolin‐7‐*O*‐glucuronide (**20**) and phenolic acids such as chlorogenic acid (**3**), and feruloyl quinic acid (**8**). The presence of flavonoids, with recognized antioxidant activities, among the chemical markers of the Prelude variety is in agreement with data published in previous work [7]. Indeed, extracts obtained from the summer variety were found to be the most active.

Different food cultivars of fennel have not been extensively reported in the literature. In 2022, Khammassi et al. studied the variability in phenolic composition in 16 populations of but only wild fennel grown in Tunisia [27], highlighting differences in phenolic production and relative antioxidant activity, which the authors attribute mainly to environmental factors.

Regarding the different plant parts of waste byproducts of cultivated fennel, previous studies by our research group have highlighted that little stems and leaves are the richest plant parts in terms of the occurrence of secondary metabolites as important antioxidants such as phenolic acids, glycosylated flavonoids and iridoids [6].

In a previous work, selected secondary metabolites from different cultivars were studied using a targeted approach. Ten polyphenolic compounds were quantified in little stems and leaves of Tiziano, Pegaso, and Preludio cultivars using the MRM (multiple reaction monitoring) method by ultra performance liquid chromatography (UPLC) coupled to QTRAP mass spectrometry. MRM showed that the Preludio cultivar was the richest in the antioxidant compounds, quercetin‐3‐*O*‐glucuronide and quercetin‐3‐*O*‐glucoside [8]. In the present paper, using a comprehensive metabolomics approach based on untargeted MS and NMR analysis, we extend the number of metabolites that can be identified as chemical markers for specific cultivars.

### 3.3 Fingerprint of *F. vulgare* Leaf and Stem Extracts by ^1^H‐NMR Analysis

To obtain a complete information on the metabolomic profile of fennel leaf and stem extracts and detect not only secondary metabolites but also primary metabolites, the extracts were investigated by ^1^H‐NMR approach. Primary metabolites are the main products of metabolic processes involved in different living functions required for plant life, such as cellular functions, growth, and reproduction; it is known their function as signal molecules to trigger defense response by signal transduction and pathogen recognition processes [28]. Therefore, to identify this kind of compounds, ^1^H‐NMR analysis of fennel leaf and little stem extracts was achieved (Figure S1). By ^1^H‐NMR, a key resonance was assigned for each metabolite, and 15 primary metabolites were identified. In particular, in the region of amino acids, isoleucine (δ 0.93, t, *J* = 7.2 Hz), valine (δ 1.05, d, *J *= 7.0 Hz), threonine (δ 1.23, d, *J* = 6.6 Hz), alanine (δ 1.46, d, *J* = 7.0 Hz), lysine (δ 1.50, m), aspartic acid (δ 2.74, dd, *J* = 3.7, 16.5 Hz), proline (δ 1.98, m), phenylalanine (δ 7.31, m), and betaine (δ 3.24, s) were assigned [29]. In the carbohydrates region, the ^1^H‐NMR spectra presented specific signals assigned to *β*‐glucopyranose (δ 4.50, d, *J* = 8.0 Hz), *α*‐glucopyranose (δ 5.13, d, *J* = 3.8 Hz), and sucrose (δ 5.40, d, *J* = 3.8 Hz). Primary metabolites such as amino acids and carbohydrates do not have direct pharmacological effects but are crucial for maintaining overall health and facilitating the proper functioning of the body [28, 30]. Among primary metabolites, some compounds are recognized as functional ingredients [31]. In detail, betaine is well‐known as a healthy ingredient for its capacity to attenuate oxidative stress, endoplasmic reticulum stress, inflammation, and cancer development [32, 33, 34, 35].

**FIGURE 3:**
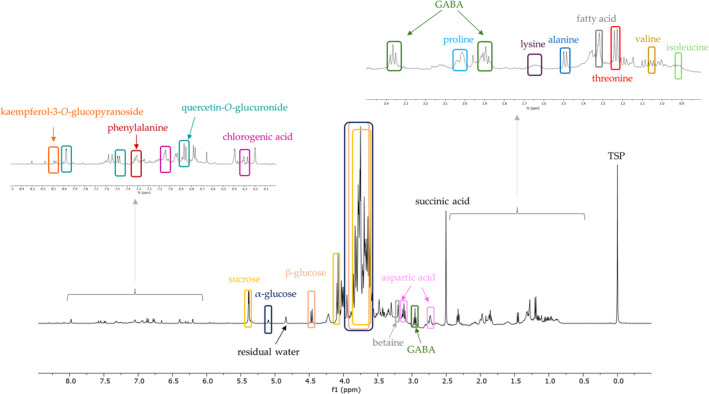
^1^H‐NMR spectrum of Preludio *Foeniculum vulgare* leaf extract.

In the same region, characteristic signals at δ 1.92 (t, *J* = 7.5 Hz), 2.36 (t, *J* = 7.2 Hz), and 3.0 (t, *J *= 7.5 Hz) were recognized as *γ*‐aminobutyric acid (GABA). GABA is a naturally occurring potential bioactive present in plants, microorganisms, animals, and humans. As a main inhibitory neurotransmitter in the central nervous system, GABA possesses a broad spectrum of promising bioactivities. A recent review summarized the various health benefits of GABA‐enriched foods, mainly including neuroprotection, anti‐insomnia, antidepression, antihypertensive, antidiabetes, and anti‐inflammatory [36]. Finally, the signal at δ 2.53 (s) was attributed to succinic acid [37] (Figure 3 and Table S1), an organic acid reported for the improvement of motor behavior and amelioration of cognitive deficits. It mitigated mitochondrial oxidative phosphorylation dysfunction in animal models of neurodegenerative diseases and improved the metabolic profile in high‐fat diet‐induced obesity [31]. Moreover, the careful analysis of ^1^H‐NMR data of Preludio leaf extracts allowed us to identify key signals for three of the main secondary metabolites present in the extract; in detail, the comparison with reference standards permitted us to unambiguously assign peaks at δ 8.05 (d, *J* = 8.0 Hz), 6.86 (d, *J* = 8.0 Hz), and 6.33 (d, *J* = 16.0 Hz) that were assigned to kaempferol‐3‐*O*‐glucopyranoside, quercetin‐*O*‐glucuronide, and chlorogenic acid, respectively (Figure 3).

### 3.4 Pseudo‐Targeted Multivariate Statistical Analysis of Primary Metabolites by ^1^H‐NMR Analysis

PCA was performed to explore the differences in the occurrences of primary metabolites among the various samples. In this case, a matrix characterized by the area of the peak identified by ^1^H NMR analysis (variables) and by different extracts (observations) corresponded to rows and columns of the matrix, respectively. The resulting model corroborated the significance and predictability of the model (PC1 contributed to 75.0% of the variance, and PC2 contributed to 12.0%).

**FIGURE 4:**
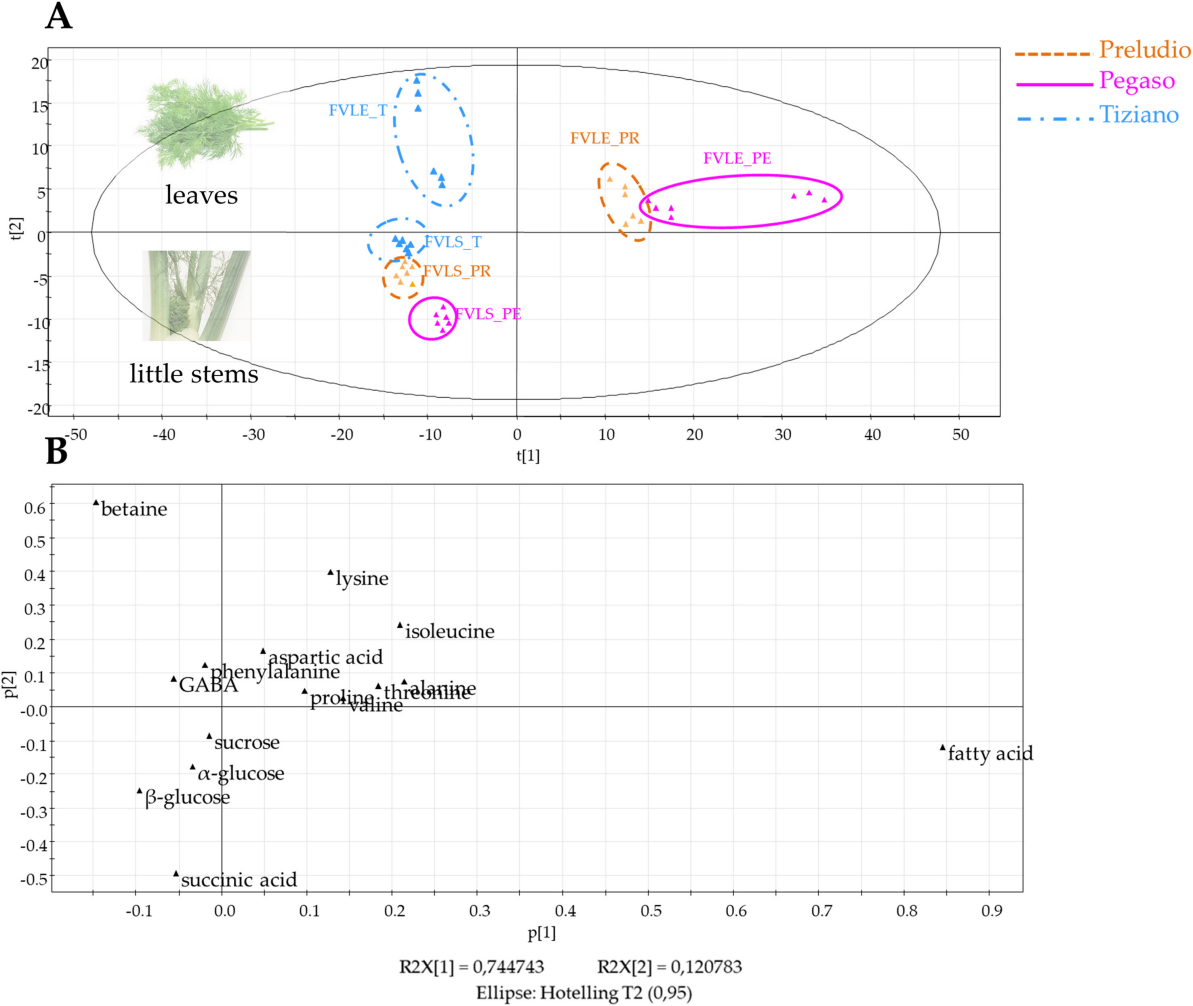
PCA score scatter plot (A) and loading scatter plot (B) of primary metabolites in fennel extracts (little stems and leaf) by ^1^H‐NMR Pseudo‐Targeted Multivariate Statistical Analysis.

PCA scores plot of the ^1^H‐NMR spectral data indicated only the differences between plant parts rather than between varieties. As observed in Figure 4A, little stem extracts on the lower quadrants were separated from the extracts of the leaf extracts on the upper quadrants through the second principal component. The PCA loadings plot (Figure 4B) allowed us to identify the metabolites responsible for the separation. In detail, stem extracts were richer in carbohydrates (*α*‐glucose, *β*‐glucose, and sucrose), succinic acid, and fatty acid, while leaf extracts were richest in amino acids, betaine, and GABA.

**FIGURE 5:**
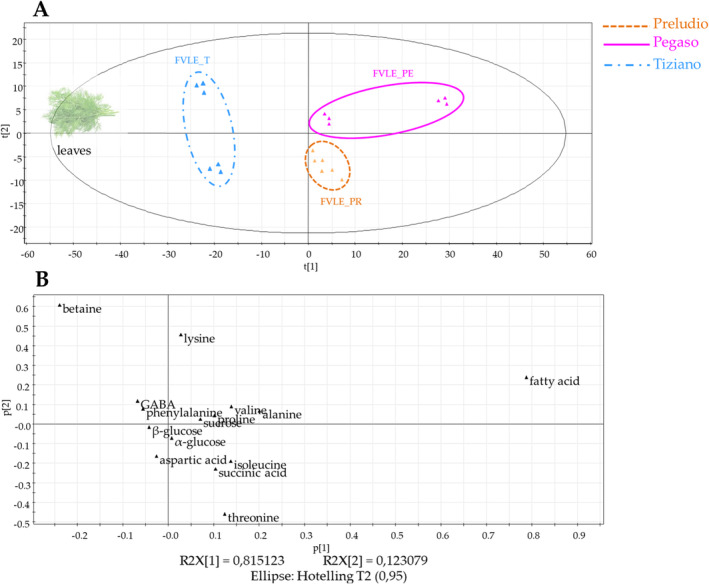
PCA scores (A) and loadings (B) scatter plots of ^1^H‐NMR spectral data for primary metabolites in fennel leaf extracts determined by pseudo‐targeted multivariate statistical analysis.

With the aim to highlight if stem and leaf extracts were separated according to the variety, PCA‐based ^1^H‐NMR metabolomics, separately of leaf and stem extracts, was performed; in both cases, the extracts showed, on PCA score plot, a distribution depending on varieties (Figure 4, Figure S2). Fennel leaves are used in traditional medicine for a wide range of applications regarding mainly digestive apparatus (gastralgia, abdominal pain, gastritis, and diarrhea) and anti‐inflammatory effects (arthritis and fiver) [5]. Our previous study on fennel byproducts highlighted that the leaf extracts contain the highest content of phenolic and flavonoids responsible of the bioactivity associated to this byproduct [7, 8, 38]; therefore, herein, our attention was focused only on the leaf extracts of the Preludio, Tiziano and Pegaso varieties. A matrix characterized by the area of the peak identified by ^1^H NMR analysis (variables) and by different extracts (observations) corresponding to rows and columns of the matrix, respectively, was used. Pareto scaling was applied before PCA; the matrix was built by 15 variables (specific chemical shift of primary metabolites) and 6 observations (leaf extracts). The PCA scores plotted in Figure 5A showed an evident separation among the samples, in distinct quadrants; Tiziano leaf extracts were located on the left quadrants, while Pegaso leaf samples were on the upper right quadrant and Preludio leaf samples were on the lower right quadrant. Hence, the PCA loadings plot (Figure 5B) was able to reveal the biomarker compound for each variety. Tiziano leaf samples showed a higher content of betaine, aspartic acid, phenylalanine, GABA, and *β*‐glucose. Preludio leaf samples displayed a higher occurrence of isoleucine and threonine amino acids, *α*‐glucose and succinic acid, while Pegaso leaf extracts were richest in amino acids that include alanine, lysine, proline, and valine.

### 3.5 Partial Least Squares‐Discriminant Analysis (PLS‐DA) of MS‐NMR Fused Data

**FIGURE 6:**
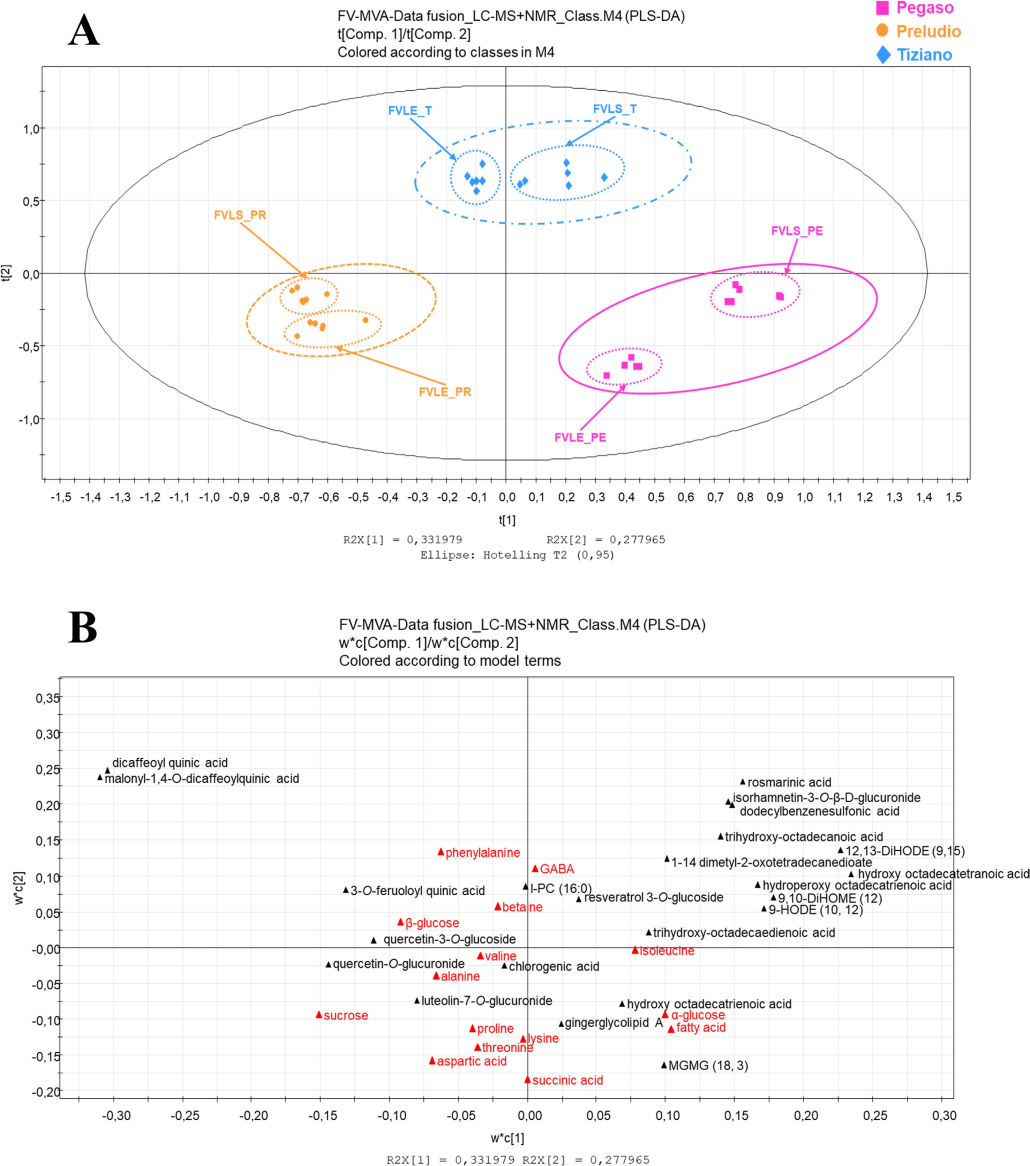
PLS‐DA scores scatter plot (A) and loading scatter plot (B) of MS‐NMR fused datasets. Primary metabolites in red and secondary metabolites in black.

The LC–MS and NMR spectral datasets were merged into a new data matrix with 36 observations, with variables representing both primary and secondary metabolites. Data transformation and scaling were significant for constructing the final matrix for multivariate data analysis. Dimensional differences between variables from two spectral datasets on two different blocks were considered. Data transformation and scaling are detailed in the experimental section. This analytical approach allowed a more global interpretation of the metabolomes of three fennel cultivars. The data matrix was subjected to a PLS‐DA analysis, differentiating the samples into three classes based on the varieties under investigation (−1, 0, +1). PLS‐DA analysis was performed to improve the visualization and interpretation of the model, and they were found to have a good fit and a satisfactory degree of predictability. The model was generated using the first and second principal components. The first component afforded a variance of 33% and the second at 28%, with a Q_2_ score of 89%. From the scores scatter plot in Figure 6A, the three varieties exhibited different metabolic expressions, as indicated by three distinct clusters. Tiziano samples were clustered in the upper quadrants, while those of the Preludio variety were in the lower left quadrant and the Pegaso variety in the lower right quadrant. In addition, the stem and leaf samples for each variety afforded a distinct subcluster, suggesting differences in primary and secondary metabolic profiles in respective plant parts.

Metabolites found as chemical markers of Tiziano are phenylalanine, GABA, betaine, *β*‐glucose (primary metabolites), and dicaffeoylquinic acid, malonyl‐1,4‐*O*‐dicaffeoylquinic acid, rosmarinic acid, isorhamnetin‐3‐*O*‐*β*‐D‐glucuronide, dedecylbenzenesulfonic acid, trihydroxy‐octadecanoic acid, 12,13‐DiHODE (9, 15), 1–14‐dimethyl‐2‐oxotetradecanedioate, hydroxy octadecatetranoic acid, hydroperoxy octadecatrienoic acid, 9,10‐DiHOME (12), 9‐HODE (10, 12), trihydroxyoctadecadienoic acid, resveratrol 3‐*O*‐glucoside, l‐PC (16:0), 3‐*O*‐feruloylquinic acid, and quercetin‐3‐*O*‐glucoside (secondary metabolites). Metabolites found as chemical markers of Pegaso are isoleucine, fatty acid, succinic acid, *α*‐glucose (primary metabolites) and hydroxyoctadecatrienoic acid, gingerglycolipid A, MGMG (3, 18) (secondary metabolites). Metabolites found as chemical markers of Preludio are lysine, proline, threonine, aspartic acid, alanine, valine, sucrose (primary metabolites), and chlorogenic acid, quercetin‐*O*‐glucuronide, and luteolin‐7‐*O*‐glucuronide (secondary metabolites). Pegasus (spring) and Preludio (summer) are separated from the Tiziano winter variety. The loadings show that this clustering is mainly due to an increased expression of primary metabolites primarily involved in plant growth and development.

The MS‐NMR data fusion model allows us to conclude that both primary and secondary metabolites participate in the differentiation of samples based on fennel variety. Additionally, the MS/NMR‐based data fusion approach was quite useful to further validate statistical models performed on spectral datasets on earlier batches of samples.

## 4 Conclusions

The three fennel varieties are indeed different in three factors:variety, geographical location, and seasonality. In fact, Pegaso and Preludio varieties were cultivated in spring and summer, respectively, while Tiziano variety is a winter crop. As can be seen, the Figure 2 shows that the summer and winter varieties are closer to each other in terms of metabolism than the spring varieties. A cluster separation, indicating differences in metabolic expression between the two plant parts, was also observed between the small stem and leaf extracts of the Pegaso and Tiziano varieties.

On the other hand, the Pegaso and Tiziano varieties are cultivated in the same region of Italy, which is different from the cultivation area of the Preludio variety. However, looking at the distribution of the varieties in the scatter plot, there does not seem to be enough commonality in the secondary metabolites to form a distinct cluster. This observation allows us to conclude that the most crucial factor in determining the differences in the metabolism of the samples analyzed is the variety. Indeed, the varieties Pegaso, Preludio, and Tiziano generate three distinct clusters in the multivariate analysis based on the profiling of their secondary metabolites. The study also suggests the possibility of growing different varieties of fennel at different times of the year as well as in other regions. Always bear in mind, however, that the different varieties can produce the bulb, which is the marketed part, in the specific growing season.

LC–MS and NMR analytical technologies were efficaciously used to distinguish metabolites expressed from different cultivars of *Foeniculum vulgare* , characterized by different seasonality.

The findings in this study validate that different cultivars respond in metabolism differently to geographical and climate factors by displaying different chemical compositions, and subsequently will result in diverse nutraceutical properties due to chemicals expressed. In fact, nutraceuticals are nutritional ingredients biologically active and have the potential to maintain optimal health and benefits. These products play a vital role in the care and management of human health, especially for future therapeutic development. Nutraceuticals have gained recognition for their nutritional benefits, therapeutic effects, and safety profile. Nutraceuticals are booming around the world in various service fields such as health promotion and disease control [39]. Since the agri‐food industry produces enormous quantities of waste byproducts, scientific research seeks to valorize them in a circular economy. The occurrence of bioactive compounds in these waste byproducts can be used to produce nutraceuticals to improve human health in the prevention and treatment of various diseases [40, 41]. By applying LC–MS and NMR‐based metabolomics combined with multivariate data analysis, it was possible to screen the chemical profile of waste byproducts coming from different fennel cultivars, and this finding will be helpful in guiding the valorization of these byproducts for commercial purposes.

## Supporting information


**Table S1** ^1^H NMR data with annotations of identified primary metabolites detected in the extract of the *Foeniculum vulgare* leaves Preludio.
**Figure S1.** ^1^H NMR spectra of the fennel extracts.
**Figure S2.** PCA score scatter plot(A) and loading scatter plot (B) of primary metabolites in little stem extracts by ^1^H‐NMR Pseudo‐Targeted Multivariate Statistical Analysis.

## Data Availability

The data used to support the findings of this study can be made available by the corresponding author upon request.
