# Supplementary material for: Comparison of Vegetable Waste Byproducts of Selected Cultivars of Foeniculum vulgare Mill. by an Integrated LC‐(HR)MS and 1H‐NMR‐Based Metabolomics Approach
Source: Phytochem Anal. 2025 Jan 21;36(4):1017–30. doi: 10.1002/pca.3488 (PMC12129718; doi:10.1002/pca.3488)
Supplement: Supplementary file 1 — Table S1 1H NMR data with annotations of identified primary metabolites detected in the extract of the Foeniculum vulgare leaves Preludio. Figure S1. 1H NMR spectra of the fennel extracts. Figure S2. PCA score scatter plot(A) and loading scatter plot (B) of primary metabolites in little stem extracts by 1H‐NMR Pseudo‐Targeted Multivariate Statistical Analysis. [file PCA-36-1017-s001.docx]

**Comparison of selected cultivars of *Foeniculum vulgare* Mill. leaves and little stems by an integrated LC-(HR)MS and ^1^H-NMR based metabolomics approach.**

**Maria Assunta Crescenzi ^1,2^, Antonietta Cerulli ^1^, Milena Masullo ^1^, Paola Montoro ^1,^* and Sonia Piacente ^1^.**

*^1^ Department of Pharmacy, University of the Study of Salerno, Via Giovanni Paolo II 132, I-84084 Fisciano, Italy*

*^2^ Ph.D. Program in Drug Discovery & Development, Department of Pharmacy, University of the Study of Saler-no, Via Giovanni Paolo II 132, I-84084 Fisciano, Italy*

*^3^  National Biodiversity Future Center (NBFC), 90133, Palermo, Italy*

******* *Correspondence: pmontoro@unisa.it*

.

*Reagents and Solvents*

Ethanol and water used for the extractions were purchased from VWR (Milan, Italy). Acetonitrile (ACN), formic acid, water and methanol of LC-MS grade were supplied by Merk (Merk KGaA, Darmstadt, Germany). Methanol *d_4_*, TPS (3-(Trimethylsilyl)-propionic-2,2,3,3-*d_4_* acid sodium salt at 0.9% (w/w) in D_2_O 99.9 %.

*LC-ESI/LTQOrbitrap/MS analysis*

LC-HRMS analysis were carried out by developing a method using an HPLC coupled with a hybrid mass spectrometer, which combines the linear trap quadrupole (LTQ) and Orbitrap mass analyzer. The experiments were executed with a Thermo scientific liquid chromatography system, equipped with a quaternary Accela 600 pump and an Accela autosampler, combined with a Linear Trap-Orbitrap hybrid mass spectrometer (LTQ-Orbitrap XL, Thermo Fisher Scientific, Bremen, Germany) equipped with an electrospray ionization (ESI) source. A Kinetex EVO 5.0 µm column (150 mm × 2.1 mm) column (Phenomenex Aschaffenburg, Germany) was used to perform the separation. The mobile phases employed were water + 0.1% formic acid (A) and acetonitrile + 0.1% formic acid (B). An increasing linear-gradient (v/v) at a flow rate of 0.200 mL/min of solvent B was used: 0–10 min, from 5 to 15%; 10–30 min, from 15 to 35%; 30–40, from 35 to 80% and then back to 5% for 10 min. The mass spectrometer operated in negative ion mode and 10 µL of each sample was used for injection. ESI source parameters were the following: capillary voltage -48 V; tube lens voltage -176.47 V; ion source temperature 280 °C; sheath and auxiliary gas flow (N2), 15 and 5; sweep gas 0; capillary voltage 3.5 kV. The full range m/z adapted to the acquisition of MS spectra was 180–1400. A collision energy of 30% was set up for the fragmentation study. Xcalibur software version 2.2 was used for instrument control, data acquisition and data analysis.

*NMR Analysis and Data Processing*

NMR analyses were carried out on a Bruker-500 spectrometer (Bruker BioSpin GmBH, Rheinstetten, Germany) equipped with a Bruker 5 mm Methanol-*d_4_* (99.95%, Sigma-Aldrich) was used as solvent for each extract.

All samples were run at 300 K, using the zgesgp pulse sequence; the relaxation delay was 4.0 s, and the acquisition time was 5.45 s, with 128 number scans and data collected into 64 k data points. Each free induction decay (FID) was zero-filled to 128 k data points. Before Fourier transformation, an exponential window function with a line broadening factor of 0.3 Hz was applied. For the HSQC analysis, a spectral window of 12 ppm and 165 ppm was used for proton and carbon, respectively, 1 K data point, 64 number scans, increments of 256 t1, and a recycle delay of 2 s. The HMBC was obtained with a spectral window of 12 ppm and 230 ppm for proton and carbon, respectively, 4 K, 120 scans, increments of 256 t1, and a recycle delay of 2 s. COSY measurements were made with 4 k × 256, a spectral width of 12 in either dimension, 24 scans, and a 2 s relaxation delay. For the carbohydrates, TOCSY measurements with a spectral width of 10 and 120 scans were acquired.

After the acquisition, ^1^H-NMR spectra were analyzed by MestreNova 10 software. In particular, the spectra were manually phased and baseline corrected. Spectra were referenced using the TSP obtaining good peak alignment. Bucketing was performed within the -0.5-8.5 ppm region (spectral buckets of 0.04 ppm), excluding the signals of the residual non-deuterated methanol, and deuterated methanol. The obtained data set was normalized on TSP value. Finally, the spectra were converted to ASCII format. Each sample was prepared in duplicate, on each single sample the experiments were repeated three times.

Pareto scaling was applied before multivariate data analysis; in detail, the matrix was constituted by 15 variables, corresponding to key chemical shifts of primary metabolites of *F. vulgare*, and 12 observations, represented by extracts.

**Table S1** ^1^H NMR data with annotations of identified primary metabolites detected in the extract of the *F. vulgare* leaves Preludio

| compound | ^1^H chemical shift  (multiplicity, *J* in Hz) |
| --- | --- |
| isoleucine | 0.93* (t, 7.2) |
| valine | 0.99 (d, 7.0), 1. 05 (d, 7.0) *, |
| threonine | 1.23 (d, 6.6) |
| fatty acid | 1.28* (s) |
| alanine | 1.46* (d, 7.0), 3.85 (q, 6.0) |
| lysine | 1.50 (m)*, 1.62 (m) |
| GABA | 1.92 (t, 7.5), 2.36* (t, 7.2), 3.0 (t, 7.5) |
| proline | 1.98 (m)*, 2.00 (m) |
| succinic acid | 2.53* (s) |
| aspartic acid | 2.74* (dd, 3.8, 16.6), 2.85(dd, 16.6,3.8) |
| betaine | 3.24 (s) |
| β-glucose | 4.50* (d, 8.0) |
| α-glucose | 5.13* (d, 3.8) |
| sucrose | 5.40 * (d, 3.8), 4.10 (d, 8.8), |
| phenylalanine | 7.31* (m) |

*
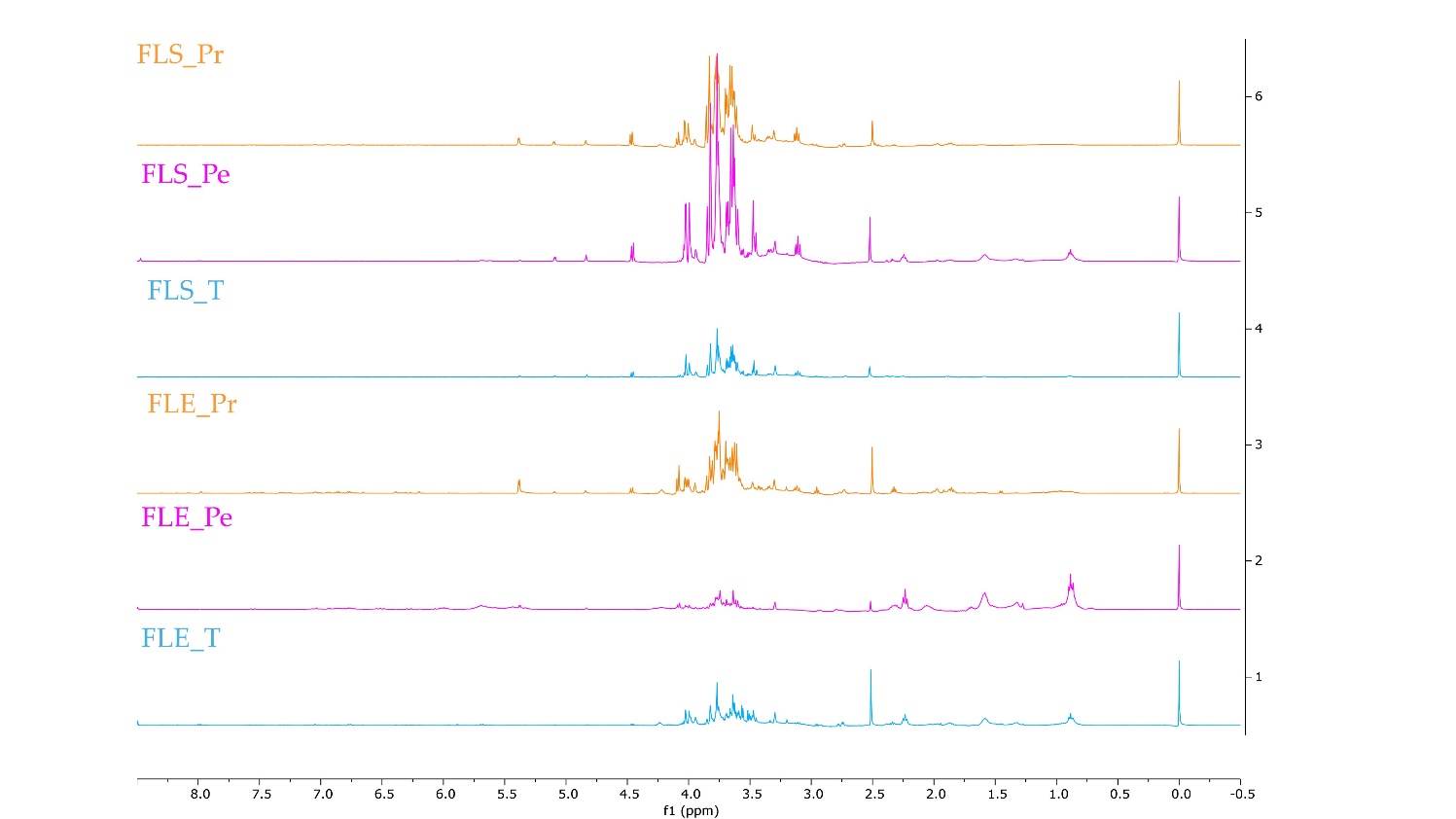
*

**Figure S1.** ^1^H NMR spectra of the fennel extracts


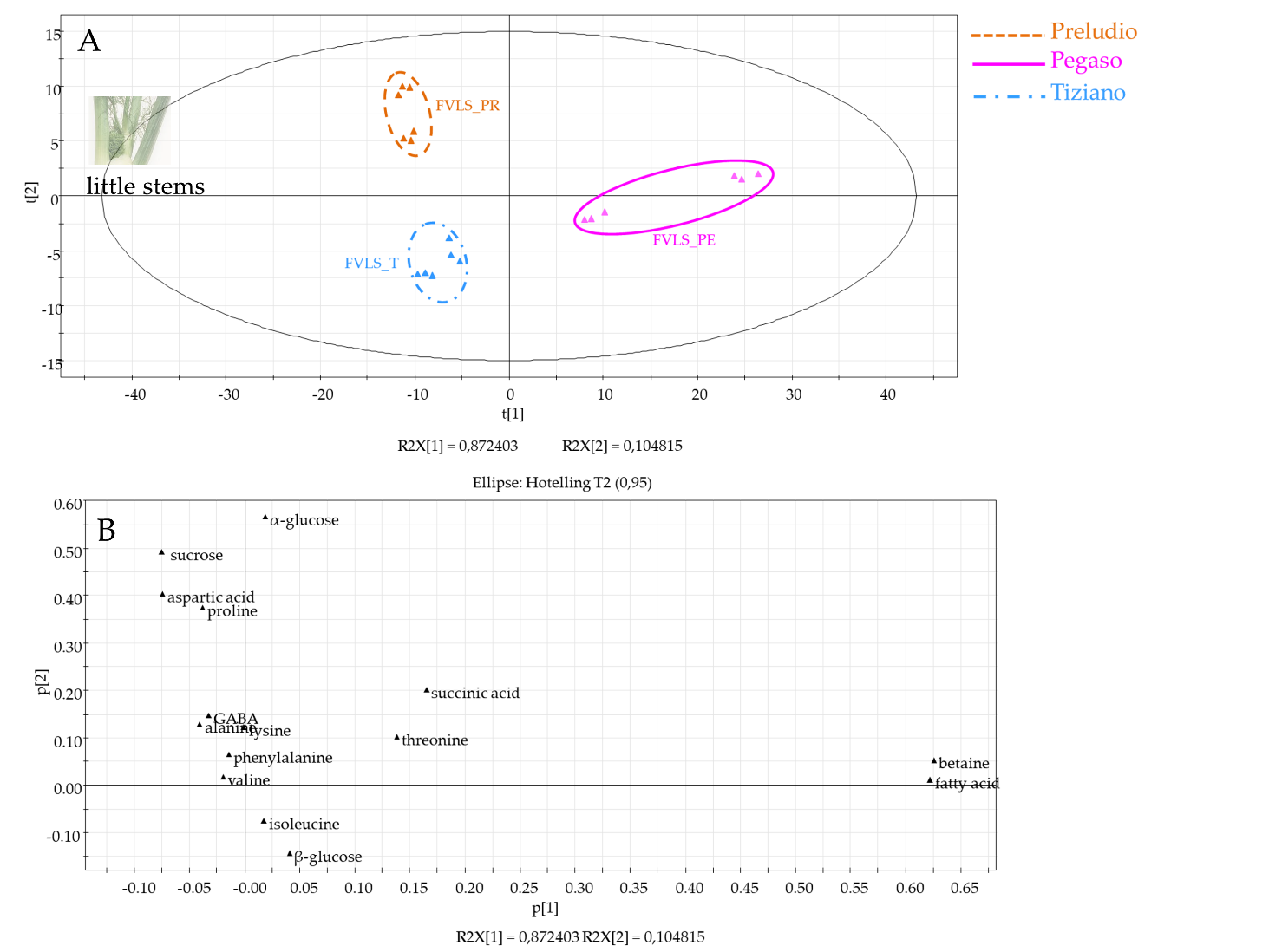


**Figure S2.** PCA score scatter plot(A) and loading scatter plot (B) of primary metabolites in little stem extracts by ^1^H-NMR Pseudo-Targeted Multivariate Statistical Analysis.
